# Mental health rehabilitation models for people with complex psychosis: a systematic review

**DOI:** 10.1186/s12888-025-07174-8

**Published:** 2025-08-26

**Authors:** Nikola Houngbedji, Christian Dalton-Locke, Amelia Hughes, Helen Killaspy

**Affiliations:** 1 Division of Psychiatry, University College London, Maple House, 149 Tottenham Court Road, London, W1T 7NF UK; 2 North London NHS Foundation Trust, St. Pancras Hospital, 4 St. Pancras Way, London, NW1 0PE UK

**Keywords:** Mental health, Complex psychosis, Rehabilitation, Model of care, Effectiveness, Systematic review

## Abstract

**Background:**

Mental health rehabilitation services support people with complex psychosis to live successfully in the community. This systematic review aimed to identify and compare the approach and effectiveness of different models of mental health rehabilitation.

**Methods:**

Six online databases (PsycINFO, EMBASE, Cochrane Library, CINAHL Plus, MEDLINE and Emcare) were searched for quantitative, qualitative, and mixed-methods studies published since 2000 that described mental health rehabilitation models. The search was extended by screening references and citations of included studies. A narrative synthesis of included studies was conducted, describing the characteristics and effectiveness of the different models.

**Results:**

We identified 24 studies which met our inclusion criteria. The studies were categorised based on the rehabilitation model that they described or the main defining feature of the rehabilitation model if it was not a named model (Strengths-Based (*n* = 4), Goal-Oriented (*n* = 2), Holistic Care (*n* = 4), Community Rehabilitation in Low-Income Countries (*n* = 4), Illness Management and Recovery Programmes (*n* = 5), Intensive Case Management (*n* = 2), and Psychosocial Rehabilitation (*n* = 3)). The rehabilitation models originated from a range of settings, including high-income (Australia, Denmark, Hong Kong, Israel, Italy, Korea, Netherlands, Spain, Sweden, United States), upper-middle-income (China), and low-income countries (Ethiopia, India, Indonesia, South Africa, Sudan, Turkey). While many of the models were shown to be effective in specific areas, such as personal recovery, symptom reduction, and social functioning, none emerged as universally superior, with varying strengths and limitations across different contexts and outcomes.

**Conclusion:**

A range of mental health rehabilitation models exists globally. Whilst some appear suited to certain contexts and some have demonstrated effectiveness in regard to specific outcomes, greater consensus on the specific components comprising a comprehensive biopsychosocial approach to mental health rehabilitation for people with complex psychosis is needed. However, a degree of flexibility is required to ensure the model can be effectively implemented in local settings.

**Review registration:**

This review was prospectively registered on PROSPERO (ID: CRD42024542294) on 2 May 2024.

**Supplementary Information:**

The online version contains supplementary material available at 10.1186/s12888-025-07174-8.

## Background

Around 20% of individuals who develop psychosis will have complex problems that impair their ability to live independently in the community [1, 2]. Most people with complex psychosis have a diagnosis of schizophrenia, schizoaffective disorder, or bipolar disorder, with treatment-resistant symptoms. These include so-called ‘positive’ symptoms, such as hallucinations and delusions, and ‘negative’ symptoms, such as apathy, amotivation, blunted affect, and cognitive impairments, which affect organisational skills and verbal memory. Often, there are additional problems that further complicate recovery, including pre-existing conditions, such as intellectual disability or developmental disorders (e.g. those on the autism spectrum) and co-existing mental and physical health conditions [3]. This is a group who are highly vulnerable, with up to 70% or so experiencing severe self-neglect and over 40% experiencing sexual or financial exploitation historically [4]. Together, these problems significantly impact individuals’ social and everyday functioning, often necessitating recurrent and/or lengthy inpatient admissions and intensive community support when discharged [5], resulting in high costs of care. It has been estimated that although they represent a relatively small proportion of all those with mental health problems, this group, people with ‘complex psychosis’, absorb around half of the total health and social care budgets for mental health [6].

Frost and colleagues in 2017 proposed the Integrated Recovery-oriented Model (IRM) for people with complex psychosis [7]. The IRM emphasised the importance of personal recovery, instilling hope, and a longer-term holistic approach to rehabilitation. In 2020, the National Institute for Health and Care Excellence (NICE), an organisation which provides evidence-based guidance to the National Health Service and the wider health and care system in the UK, published a guideline for mental health rehabilitation which also took a recovery-oriented, holistic approach [3]. The guideline recommended that people with complex psychosis should have access to local specialist rehabilitation services that should include inpatient rehabilitation units, supported accommodation services, and community rehabilitation teams. These services should form an integrated local pathway which supports individuals to gain the confidence and skills to transition from higher supported to more independent settings as they progress in their recovery. NICE recommends that rehabilitation services should operate with a recovery-orientated and optimistic culture, be staffed by multidisciplinary teams to support people across multiple domains, tailored to individual support needs, through a range of biopsychosocial interventions including optimising medication, addressing physical health issues, enabling activities of daily living (ADL) skills (personal hygiene, shopping, cooking, cleaning, budgeting, etc.), and engaging with community activities [3]. Internationally, various models of rehabilitation have been developed, each with their own focus or slightly different approach, and various studies have been conducted to evaluate their effectiveness [8].

In the UK, national research programmes on mental health rehabilitation services have shown that the recovery orientation of the service is positively associated with successful progression to more independent settings, with around two-thirds of those in inpatient rehabilitation (approximately 235 out of 329 participants) and over 40% of those in supported accommodation (243 out of 586 participants) achieving this in the expected timeframes [9, 10]. A retrospective cohort study in London also found that around two-thirds of people (83 out of 141) progressed along the care pathway as expected over a five-year period [11]. In a case-control study in Ireland, people who had access to mental health rehabilitation services (*n* = 126) were more likely to achieve successful community discharge and had greater improvements in their social functioning (OR 8.44, 95% CI 4.16–17.16) than people without access or awaiting these services (*n* = 74) [12].

Research to improve the recovery orientation of mental health rehabilitation services is also ongoing. In the Netherlands, the ‘Active Recovery Triad’ (ART) model [13] has been developed, which emphasises active engagement, recovery-focused care, and collaboration between service users, professionals, and families to achieve structured recovery goals within a defined timeframe, personal empowerment, and integration into the community. The ART model is currently being evaluated [13].

This systematic review aimed to identify existing mental health rehabilitation models used for both community and inpatient settings in the international literature. We also aimed to compare and contrast these models, both in terms of their approach and their effectiveness on a range of outcomes, including how well they support people with complex psychosis with their everyday functioning, social functioning, personal recovery, and clinical recovery.

## Methods

### Eligibility criteria

The inclusion and exclusion criteria were designed using the PICOS framework [14].

#### Population

The review included studies reporting on mental health rehabilitation models designed to support adults aged 18 and over with complex psychosis. Studies of models designed for individuals with first-episode psychosis, children and adolescents, or for people without a primary diagnosis of schizophrenia, schizoaffective disorder, bipolar affective disorder, or depression with psychosis, were excluded. Studies were also excluded if fewer than 50% of participants had a primary diagnosis of schizophrenia, schizoaffective disorder, bipolar affective disorder, or depression with psychosis.

#### Intervention

There is a large variation in how the term ‘rehabilitation’ is used in the mental health literature [15, 16]. For this review, a mental health rehabilitation model was defined as: a multi-component, biopsychosocial approach, delivered by a multidisciplinary team, that aims to improve clinical, psychological, and/or social outcomes for individuals with complex psychosis. Studies evaluating Assertive Community Treatment (ACT) - a specific form of Intensive Case Management (ICM) - were excluded on the basis that this model has been extensively reviewed, most recently by Dietrich et al. [17] and Harvey et al. in 2021 [18], and been shown to be clinically and cost-effective, but it does not specifically focus on people with complex psychosis. However, studies of ICM published after October 2021 were included, as it is less clearly defined than ACT [9], ensuring that relevant papers potentially excluded from the recent reviews of ACT were not overlooked.

#### Comparison

Studies describing or evaluating mental health rehabilitation models were eligible for inclusion, whether or not a comparison group was included.

#### Outcomes

Studies were included if they reported on at least one of the following outcomes:

- description of a mental health rehabilitation model for people with complex psychosis.
- effectiveness of a mental health rehabilitation model for people with complex psychosis, assessed through clinical outcomes (such as symptoms, and inpatient and community service use), personal recovery, social functioning, activities of daily living (for example, personal hygiene, shopping, cooking, budgeting etc.), quality of life, and satisfaction with care.
- any other reported outcomes not relating directly to the individual, including but not limited to measures of costs and cost-effectiveness.

#### Study design

The review included quantitative, qualitative, and mixed-methods studies. Non-peer-reviewed articles such as editorials, opinion articles, conference abstracts, book chapters, and trial protocols were excluded.

### Search strategy

Six electronic databases—PsycINFO, EMBASE, Cochrane Library, CINAHL Plus, MEDLINE and Emcare—were searched using free-text terms and subject terms which related to two concepts: complex psychosis and mental health rehabilitation. Limits relating to age (18 and over), human, English language and publication date (> 2000) were applied. This date was selected to enable a focus on contemporary rehabilitation models. The searches were carried out on 16 May 2024, and the results were exported to Covidence for de-duplication.

The titles and abstracts of all studies were screened in parallel. After this stage, the full texts of the studies were screened for final inclusion. The screening, using the eligibility criteria, was carried out by the lead researcher (NH), with 10% of articles at both the title/abstract and full-text stages independently screened by a second researcher (AH). The two researchers discussed any discrepancies, and any that could not be resolved were adjudicated by a third researcher (HK or CDL). All included studies after the full-text screening stage were subject to backward and forward citation searches. Please see Additional file 1 for further details of the search strategy. 

### Data extraction

A data extraction form was created to facilitate the recording of key study information relating to the review, such as study design, sample size and participant characteristics, and outcomes, including any description of the rehabilitation model.

### Quality assessment

All included studies were assessed using Kmet’s standardised quality assessment criteria for evaluating primary research studies [19]. This assessment includes 14 criteria for quantitative studies and 10 criteria for qualitative studies. These criteria relate to the study design, participant selection, data analysis methods, clarity and interpretation of results. Each criterion was scored as ‘fully met’ (= 2), ‘partially met’ (= 1), and ‘not met’ (= 0). A % score for each paper was calculated by summing the scores of applicable items and dividing this by the total possible score, and then multiplying this number by 100. The assessment was conducted alongside the data extraction process by the lead researcher (NH). A second researcher (AH) independently assessed the quality of 20% of the studies.

### Data synthesis

A meta-analysis was initially considered; however, due to the heterogeneity in rehabilitation models, study designs, and outcomes of the included studies, a narrative synthesis was the most appropriate method to summarise the findings. The synthesis was structured using the guidelines published by Popay et al. [20]. Stage 1 *(Developing a Theory)* was not applicable, as we aimed to compare existing models rather than generate new theoretical explanations. In Stage 2 (*Developing a Preliminary Synthesis*), we began by writing a brief description of each of the rehabilitation models. We then compared these models, whilst considering whether they could be grouped based on their theoretical approach and/or key components. Once all the models had been grouped, we returned to the original descriptions of these models in the included studies to check that the groupings had face value and could not be more appropriately allocated to another group, or a new group on its own if there were no other similar models. In Stage 3 (*Exploring Relationships*), we then considered the effectiveness of these models, where effectiveness had been evaluated. The weight we placed on any reported findings was informed by the study design, comparison group, sample size and characteristics, follow-up duration, and the Kmet quality score. The findings within each group were then synthesised based on the type of outcome (e.g. clinical recovery, personal recovery, etc.), consistency in direction of results, country, and setting (inpatient, residential, or community). Finally, in Stage 4 (*Assessing Robustness*), we compared the effectiveness between groups by considering the strength of the evidence for each model in terms of outcome, country, and setting. For example, model A was more effective in inpatient settings for improving personal recovery, whereas model B was the most effective model on the same outcome in community-based settings. The synthesis was led by NH but discussed throughout with AH, CDL, and HK to check for consistency in interpretation.

## Results

**Fig. 1:**
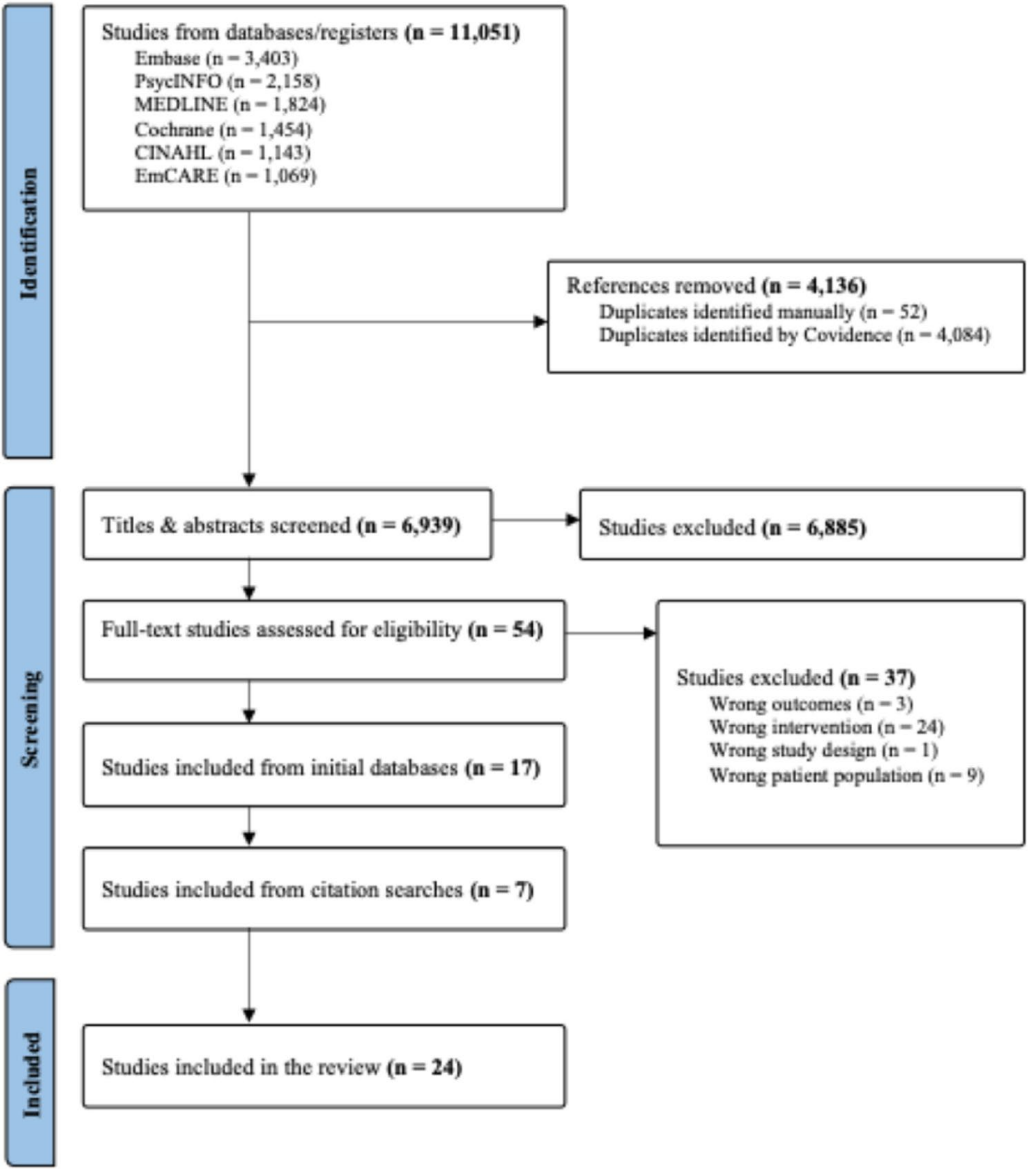
PRISMA flow diagram

The database searches yielded 11,051 studies which was reduced to 6,939 after de-duplication. Screening of titles and abstracts excluded 6,885 studies, and a full-text review of the remaining 54 led to the exclusion of another 37 studies, primarily due to not meeting the criteria for a rehabilitation model or focusing on ineligible populations. This resulted in 17 studies being included from the database searches. An additional seven studies were identified through reviewing reference lists and citation screening of these 17 studies, bringing the total number of included studies to 24. The PRISMA flow diagram for the search is shown in Fig. 1.

Of the 24 included studies, 22 were quantitative and two were qualitative in design. Of the 22 quantitative studies, the vast majority were experimental in design, including randomised controlled trials (*n* = 14), quasi-experimental studies (*n* = 2), and a non-randomised controlled trial (*n* = 1), with the remaining studies being observational (*n* = 3 prospective and *n* = 2 retrospective). The mean Kmet score for the quantitative studies was 94%, with scores ranging from 82 to 100%. The maximum score of 100% was achieved by seven studies. For the qualitative studies, one study scored 90%, and the other scored 95%. 

The included studies were conducted across a diverse range of countries, 14 studies originated from high-income countries (Australia (*n* = 1), Denmark (*n* = 1), Hong Kong (*n* = 1), Israel (*n* = 2), Italy (*n* = 1), Korea (*n* = 1), Netherlands (*n* = 3), Spain (*n* = 1), Sweden (*n* = 1), and the United States (*n* = 2)), four studies originated from the same upper-middle-income country (China), and six studies originated from low-income countries (Ethiopia (*n* = 1), India (*n* = 1), Indonesia (*n* = 1), South Africa (*n* = 1), Sudan [1], and Turkey (*n* = 1)), country income level as classified by the World Bank [21]. Most of the studies were based on community settings (*n* = 20), but a few were based on supported accommodation (*n* = 2) and inpatient settings (*n* = 1). One study explored a combination of inpatient, community, and supported accommodation settings.

Most studies focused on participants with schizophrenia, schizoaffective disorder or bipolar disorder. The majority of study participants were male. The mean age varied from 30 to 60 years.

The studies explored a diverse array of rehabilitation models and reported on a range of individual-level outcomes spanning clinical (symptoms, inpatient admission, length of hospital stay), functional (social, occupational), and personal (hope, self-efficacy, empowerment, life satisfaction, quality of life, and the internalisation of stigma) recovery. The two qualitative studies investigated patient perspectives on the models.

The tools used to assess outcomes also varied and included widely used standardised measures, subscales or specific items from these standardised tools, and custom-developed measures tailored to individual studies. Table 1 provides further details on the characteristics of the included studies.

**Table 1:** Details of the individual studies within the included mental health rehabilitation models

First author (year published)	Country	Study setting	Type of study (quantitative, qualitative, mixed methods)	Study design	Participants *N* (Intervention group = IG & control group = CG)	Diagnostic breakdown; Mean Age (SD); Gender Male %	Intervention/Comparison	Follow-up	Outcome/measures	Main Outcomes: Effectiveness of Models	Quality rating
Ahmed et al. (2021) [22]	Sudan	Inpatient setting	Quantitative	Quasi-experimental pre-post-follow-up design for one group	*N* = 49	Schizophrenia; Mean age 31.16 ± 5.12; male 49 (100%)	Social and Cognitive Rehabilitation Program	9 months	Positive and Negative Syndrome Scale (PANSS) Clifford Modified Scale Mini Mental State Examination (MMSE)​	Significant improvement in psychopathology (*p* = 0.013 post, *p* = 0.04 follow-up), social skills and cognitive function	95%
Arslan et al. (2014) [23]	Turkey	Community outpatient services	Quantitative	2-arm parallel group controlled clinical trial	*N* = 104 (IG = 55; CG = 45)	Schizophrenia; mean age IG = 40.5 ± 9.1 CG = 41.5 ± 11.5; male 71 (71%)	Psychosocial Rehabilitation Program + Medication/Medication only	6 months	Positive and Negative Syndrome Scale (PANSS); Quality of Life Scale (QLS); Social Functioning Scale (SFS); Schedule for Assessing the Three Components of Insight (SATCI); World Health Organization Disability Assessment Schedule II (WHODAS II)	Significant improvement in psychopathology, quality of life, social functioning, and insight (all *p* < 0.001), with a reduction in disability (*p* = 0.018).	95%
Asher et al. (2022) [24]	Ethiopia	Community outpatient services	Quantitative	2-arm parallel group cluster RCT, computerised randomisation 1:1 Single-centre, single-blinded	*N* = 166 (IG = 79; CG = 87)	Schizophrenia, Schizoaffective or schizophreniform disorder; mean age IG = 30 (25–45); CG = 33 (25–40); male 103 (62.05%)	Community-based rehabilitation (RISE) + Facility-based care/Facility-based care	12 months	WHO Disability Assessment Schedule (WHODAS)	Significant improvement in disability at 12 months (mean difference: − 8.13, *p* = 0.039)	100%
Brooke-Sumner et al. (2017) [25]	South Africa	Community outpatient services	Qualitative	In-depth individual interviews	*N* = 6	Schizophrenia; mean age < 45; male 66.67%	PRogramme for Improving Mental health carE (PRIME) South Africa programme	N/A	N/A	Improved self-esteem, social support, symptoms, reduced social isolation	90%
Chatterjee et al. (2003) [26]	India	Community outpatient services	Quantitative	Prospective cohort study	*N* = 207 (IG = 127; CG = 80)	Chronic schizophrenia, Paranoid illness; mean age 38.1; 36,6; male 61%	Community-based rehabilitation model (CBR)/Outpatient care (OPC)	12 months	Positive and Negative Syndrome Scale (PANSS) World Health Organization Disability Assessment Schedule (DAS)	Significant improvement in psychopathology and disability(all *p* < 0.001)	100%
Cheng et al. (2020) [27]	China	Community outpatient services	Quantitative	Longitudinal, prospective cohort study- 2-arm parallel group	N 128 (IG = 68; CG = 60)	Schizophrenia (77.3), Other (22.7%); IG = mean age 42.07 ± 9.5; CG = 41.34 ± 9.9; male 60 (47%)	Strengths Model of case management/Care as Usual (CAU)	12 months	Positive and Negative Syndrome Scale (PANSS) Personal and Social Performance (PSP) Scale Social Function 36 Scale (SF36) Stigma Self-assessment Scale	No difference between groups- endpoint: Relapse Rate: CM 7.35%, CAU Group: 5% (*p* = 0.73); PANSS (*p* = 0.95), PSP (*p* = 0.92), social functioning/quality of life (*p* = 0.97), stigma (*p* = 0.59)	96%

First author (year published)	Country	Study setting	Paradigm (quantitative, qualitative, mixed methods)	Study design	Participants *N* (Intervention = IG & control = CG)	Diagnostic breakdown; Mean Age (SD); Gender Male %	Intervention/Comparison	Follow-up	Outcome/measure	Main Outcomes: Effectiveness of Models	Quality rating
Dalum et al. (2018) [28]	Denmark	Community outpatient services	Quantitative	2-arm parallel group RCT multicentre, single -blinded, block randomisation stratified by diagnosis and CMHC to IMR + CAU or CAU.	*N* = 198 (IG = 99; CG = 99)	Schizophrenia, bipolar disorder; mean age IG = 41 ± 11.0; CG = 45 ± 11.5; male 109 (55%)	Illness Management and Recovery programme (IMR)/Treatment as usual (TAU)	9 months	Illness Management and Recovery Scales (IMRS) Adult State Hope Scale Mental Health Recovery Measure Clients Satisfaction Questionnaire	No significant differences were observed in illness management as rated by patients (*p* = 0.14) and staff (*p* = 0.76), hope (*p* = 0.53), personal recovery (*p* = 0.91), or satisfaction with treatment (*p* = 0.78).	89%
Dissanayake et al. (2024) [29]	Australia	Community outpatient services	Qualitative	Descriptive phenomenological approach​, in-depth, semi-structured interviews	*N* = 6	Bipolar Affective Disorder (BAD): 2 Psychosis: 1 Post-Traumatic Stress Disorder (PTSD): 1 Schizoaffective + BAD: 1 Unknown: 1, mean age- 29.5; 3 male and 3 female	Strengths Model of case management (SMCM)	N/A	N/A	Importance of client–case manager relationship, strengths assessment (guides personalised interventions), recovery and goals achievement	95%
Färdig et al. (2011) [30]	Sweden	Community outpatient services	Quantitative	2-arm parallel group RCT, multicentre, single-blinded, block randomisation stratified by diagnosis and clinic to IMR + CAU or CAU.	*N* = 41 (IG = 21, CG = 20)	Schizophrenia, Schizoaffective Disorder; IG = mean age 40.38 ± 6.76 years; CG = 40.45 ± 6.44 years; male 22 (53.7%)	Illness management and recovery programme (IMR)/Treatment as usual (TAU)	21 months	Illness Management and Recovery Scale (IMRS) Psychosis Evaluation Tool for Common Use by Caregivers (PECC) Manchester Short Assessment of Quality of Life (MANSA) Ways of Coping Questionnaire (WCQ) Recovery Assessment Scale (RAS) Suicidality (assessed with a modified PECC suicidality subscale) Hospitalization rates Insight (assessed with the insight subscale of the PECC)	Significant improvement in illness management and psychiatric symptoms (*p* < 0.001), positive symptoms (*p* = 0.009), negative symptoms (*p* < 0.001), depression-anxiety symptoms (*p* = 0.015), insight (*p* = 0.002), suicidal ideation (*p* = 0.013), coping strategies: seeking social support (*p* = 0.005), escape-avoidance (*p* < 0.001), planful problem-solving (*p* < 0.001), not significant: quality of life, recovery perception (RAS) (*p* > 0.05), hospitalisation (*p* > 0.05)	89%

First author (year published)	Country	Study setting	Paradigm (quantitative, qualitative, mixed methods)	Study design	Participants *N* (Intervention = IG & control = CG)	Diagnostic breakdown; Mean Age (SD); Gender Male %	Intervention/Comparison	Follow-up	Outcome/measures	Main Outcomes: Effectiveness of Models	Quality rating
Fernandez-Miranda et al. (2022) [31]	Spain	Community outpatient services	Quantitative	Observational, longitudinal, Prospective cohort study	*N* = 688 (IG = 344; CG = 344)	Schizophrenia; mean age 43.4 ± 11.4; male 427 (62.2%)	Case- managed program (CMP)/Care as Usual (CAU)	10 years	Clinical Global Impression-Severity (CGI-S) scale Number of hospitalizations Number of suicide attempts Treatment adherence	Significant reduction in psychopathology (*p* < 0.005), hospitalisation, fewer suicide attempts, treatment adherence (all *p* < 0.0001)	95%
Gelkopf et al. (2016) [32]	Israel	Community outpatient services	Quantitative	2-arm parallel group RCT; Multicentre, single-blinded, computer-based randomisation, stratified by age and service dependence, to SBCM-PRS or TAU-PRS	*N* = 1545 (IG = 808; CG = 737)	Schizophrenia, schizoaffective, and psychotic disorders 81.05%; mean age 39.2 (12.6); male 919 (59.8%)	Strengths-Based Case Management + Psychiatric Rehabilitation Services (SBCM-PRS)/Treatment as usual + Psychiatric Rehabilitation Services (TAU-PRS)	20 months	Manchester Short Assessment of Quality of Life (MANSA) Goal Attainment Scaling (GAS) Unmet Needs Scale (constructed by the research team) Self-Efficacy Scale (designed by the research team based on literature) Interpersonal Relationships Scale (based on MANSA) Colorado Symptom Index (CSI)	Significant improvement in quality of life (*p* < 0.01), self-efficacy (*p* < 0.001), unmet needs (*p* < 0.05), less decline in satisfaction with interpersonal relationships (*p* < 0.001), service utilisation (*p* < 0.001), goal attainment, no significant improvement in psychopathology	89%
Hasson-Ohayon et al. (2007) [33]	Israel	Community outpatient services	Quantitative	2-arm parallel group RCT; Multicentre, single-blinded, lottery-based randomisation to IMR or TAU	*N* = 210 (IG = 119; CG = 91)	IG = Schizophrenia: 95 (80%); CG = Schizophrenia: 81 (89%); IG = mean age 33.92 ± 11.10; CG = 35.45 ± 11.24; male 137 (65%)	Illness management and recovery programme/Treatment as usual (TAU)	8 months	Illness Management and Recovery Scale (IMRS) Coping Efficacy Scale (CES) Multidimensional Scale of Perceived Social Support (MSPSS)	Significant improvement in psychopathology, knowledge, goals factor (*p* < 0.01), clinician-rated coping factor (*p* < 0.05), no significant improvement in perceived social support	82%
Li et al. (2018) [34]	China	Community outpatient services	Quantitative	2-arm parallel group RCT; multicentre, stratified cluster randomisation	*N* = 327 (IG = 199, CG = 185)	Schizophrenia; mean age IG = 40.21 (7.57); CG = 39.70 (7.83); male 197 (51%)	Community-based comprehensive intervention/Face to face interview	9 months	Internalized Stigma of Mental Illness Scale (ISMI) Discrimination and Stigma Scale (DISC-12) Global Assessment of Functioning (GAF) Schizophrenia Quality of Life Scale (SQLS) Self-Esteem Scale (SES) Brief Psychiatric Rating Scale (BPRS) Positive and Negative Syndrome Scale for Schizophrenia - Negative Syndrome subscale (PANSS-N) Medication Compliance Assessment, Insight Assessment	Significant reduction in psychopathology, improved social functioning (both *p* < 0.001), reduced internalised stigma and discrimination (*p* < 0.05), no significant difference between groups in quality of life, medication compliance and insight (both *p* > 0.05)	86%

First author (year published)	Country	Study setting	Paradigm (quantitative, qualitative, mixed methods)	Study design	Participants *N* (Intervention = IG & control = CG)	Diagnostic breakdown; Mean Age (SD); Gender Male %	Intervention/Comparison	Follow-up	Outcome/measures	Main Outcomes: Effectiveness of Models	Quality rating
Mueser et al. (2010) [35]	United States	Community outpatient services	Quantitative	2-arm parallel group RCT; Multicentre, single-blinded, computer-based randomisation, stratified by diagnosis (mood disorder or schizophrenia and gender	*N* = 183 (IG = 88; CG = 95)	Schizophrenia: 51 (27.9%) Schizoaffective: 52 (28.4%) Depression: 44 (24.0%) Bipolar: 36 (19.7%); mean age 60.17 (7.92), male 77 (42.1%)	Helping Older People Experience Success (HOPES)/Treatment as Usual (TAU)	24 months	University of California at San Diego Performance-Based Skills Assessment (UPSA) Multnomah Community Ability Scale (MCAS) Social Behaviour Schedule (SBS) Independent Living Skills Survey (ILSS) Revised Self-Efficacy Scale (RSES) Scale for the Assessment of Negative Symptoms (SANS)	Significant improvement in psychopathology, social skills, psychosocial functioning, self-efficacy (all *p* < 0.05), community functioning (*p* < 0.01)	93%
Pelizza et al. (2023) [36]	Italy	Community outpatient services	Quantitative	Retrospective cohort study	*N* = 137	Schizophrenia/other psychotic disorders (70.1%) Bipolar disorder (17.5%) MDD with psychotic features (12.4%), mean age 32.74 ± 11.15; male 85 (62.0%)	Personal Health Budget (PHB)	24 months	Brief Psychiatric Rating Scale (BPRS) Global Assessment of Functioning (GAF) Health of the Nation Outcome Scale (HoNOS)	Significant improvements in psychiatric symptoms, particularly in negative symptoms, social functioning, and overall functioning (all *p* < 0.001).	100%
Puspitosari et al. (2019) [37]	Indonesia	Community outpatient services	Quantitative	2-arm parallel group a quasi-experimental study	*N* = 100 (IG = 50, CG = 50)	Schizophrenia, mean age 39 years; male 65 (65%)	Community-based rehabilitation (CBR)/Routine outpatient care	16 weeks	Lehman’s Quality of Life Interview (QOLI) Positive and Negative Syndrome Scale (PANSS)	Significant improvement in quality of life (*p* < 0.05); no difference between groups in psychiatric symptoms	100%

First author (year published)	Country	Study setting	Paradigm (quantitative, qualitative, mixed methods)	Study design	Participants *N* (Intervention = IG & control = CG)	Diagnostic breakdown; Mean Age (SD); Gender Male %	Intervention/Comparison	Follow-up	Outcome/measures	Main Outcomes: Effectiveness of Models	Quality rating
Roosenschoon et al. (2021) [38]	Netherlands	Supported accommodation	Quantitative	2-arm parallel group RCT; multicentre, single-blinded, 3:2 block randomization stratified by treatment teams to IMR + CAU or CAU.	*N* = 187 (IG = 116, CG = 71)	Psychotic disorders: 106 (57%) Mood disorder: 61 (33%) Personality disorders: 58 (31%), mean age 44.3 ± 10.4; male 53%	Illness Management and recovery (IMR) + care as usual (CAU)/Care as usual (CAU)	18 months	Illness Management and Recovery (IMR) Scale Coping Self-Efficacy Scale (CSES) Multidimensional Scale of Perceived Social Support (MSPSS) Service Engagement Scale (SES) Addiction Severity Index (ASI) Insight Scale Brief Symptom Inventory (BSI) Social Functioning Scale (SFS) Mental Health Recovery Measure (MHRM) Self-Esteem Rating Scale-Short Form (SERS-SF) Internal Stigma of Mental Illness Scale (ISMI)	Significant improvement in illness self-management (*p* = 0.048), self-esteem (*p* = 0.01); no significant difference in hospitalisation rates, social support, coping, medication adherence, insight, addiction between groups	93%
Salyers et al. (2014) [39]	United States	Community outpatient services	Quantitative	2-arm parallel group RCT; multicentre, single-blinded, randomisation to IMR or problem-solving group.	*N* = 118 (IG = 60; CG = 58)	Schizophrenia, Schizoaffective disorder, mean age 47.7 ± 8.9, male 94 (80%)	Illness Management and recovery (IMR)/Problem-Solving (PS) Control Group	18 months	Positive and Negative Syndrome Scale (PANSS) Quality of Life Scale (QLS) Illness Management and Recovery Scale (IMRS) Patient Activation Measure (PAM) Morisky Scale Recovery Assessment Scale (RAS) State Hope Scale	No significant differences were found between groups in psychopathology, functioning, illness management, service utilisation	93%
Sohn et al. (2023) [40]	Korea	Community outpatient services	Quantitative	Retrospective cohort study	*N* = 759	Psychotic disorder: 381 (50.20%) Mood disorder: 378 (49.80%)​; mean age 44.42 years; male 349 (46%)	Intensive Case Management program (S-ICM))	9 months	Average Length of Hospital Stay	Hospital stays reduced from 1.47 to 0.26 days/month (*p* < 0.05); sustained reduction post-intervention.	100%

First author (year published)	Country	Study setting	Paradigm (quantitative, qualitative, mixed methods)	Study design	Participants *N* (Intervention = IG& control = CG)	Diagnostic breakdown; Mean Age (SD); Gender Male %	Intervention/Comparison	Follow-up	Outcome/measures	Main Outcomes: Effectiveness of Models	Quality rating
Swildens et al. (2011) [41]	Netherlands	Inpatient care, outpatient care and/or sheltered living	Quantitative	2-arm parallel group RCT; Multicentre, single-blinded, stratified block randomisation	*N* = 156 (IG = 80; CG = 76)	Schizophrenia: 50% Schizoaffective: 15% Bipolar Disorder: 20%, MDD: 10% Other: 5%; mean age IG = 46.5 ± 12.2 years; CG = 46.3 ± 12.7 years; male 76 (48.7%)	Boston Psychiatric Rehabilitation Approach​ (PR)/Care as usual (CAU)	24 months	World Health Organization Quality of Life (WHOQOL-BREF), Camberwell Assessment of Needs, Social Functioning Scale, Personal Empowerment Scale Goal attainment- binary scale	Significant improvement in achieving personal rehabilitation goals at 24 months (adjusted risk difference: 21%, *p* < 0.05) and societal participation (*p* = 0.01); no significant differences in quality of life, social functioning, unmet needs	93%
Tao et al. (2012) [42]	China	Community outpatient services	Quantitative	Prospective Controlled Trial	*N* = 142 (IG = 90, CG = 52)	Schizophrenia; mean age IG = 40.4 (10.0), CG= 43.7 (10.4), male 72 (50.7%)	Sunshine Heart Garden program/Standard community services	12 months	Positive and Negative Syndrome Scale (PANSS) Morningside Rehabilitation Status Scale (MRSS)	Significant improvement in psychopathology, social functioning (both *p* < 0.001), no significant differences in hospitalisation rates (*p* = 0.074)	100%
Tsoi et al. (2019) [43]	Hong Kong	Supported Accommodation	Quantitative	2-arm parallel group nonrandomized Controlled Trial	*N* = 147 service users and 43 caseworkers initially (IG = 73 service users and 23 caseworkers, CG = 74 service users and 20 caseworkers)	Schizophrenia: 85% Schizoaffective: 5% Bipolar Disorder.: 5% MDD: 3% Other Psychiatric Diagnoses: 2%”, mean age IG= 46.86 (12.90%), CG= 47.51 (13.71%)male 72 (50.7%)	Strengths model of case management (SMCM)/Treatment as Usual (TAU)	12 months	Maryland Assessment of Recovery in People with Serious Mental Illness (MARS) Satisfaction with Life Scale (SWLS) State Hope Scale (SHS) Brief Psychiatric Rating Scale (BPRS) Working Alliance Inventory (WAI) Goal achievement ratings	Significant improvements in goal setting and higher goal attainment rates (*p* < 0.01). No significant difference in symptom severity, recovery, satisfaction with life, hope, well-being, work alliance (all *p* > 0.05)	100%

First author (year published)	Country	Study setting	Paradigm (quantitative, qualitative, mixed methods)	Study design	Participants *N* (Intervention = IG& control = CG)	Diagnostic breakdown; Mean Age (SD); Gender Male %	Intervention/Comparison	Follow-up	Outcome/measures	Main Outcomes: Effectiveness of Models	Quality rating
Van Busschbach et al. (2002) [44]	Netherlands	Community outpatient services	Quantitative	Prospective cohort study- Naturalistic	*N* = 35	60% schizophrenia, 17% affective disorder, 14% personality disorder, mean age 35 years (range 21–51), male 57%	Centre for Individual Rehabilitation and Education (CIRE)	12 months	Verona Service Satisfaction Schedule (VSSS-32) Camberwell Assessment of Need (CAN) EuroQoL Global Assessment of Functioning (GAF)	Goal Attainment: 46% fully achieved, 34% partly, 14% expected to; significant reductions in needs for daily activities, accommodation, social contacts (*p* < 0.05); no significant improvement in quality of life or overall functioning.	95%
Wang et al. (2013) [45]	China	Community outpatient services	Quantitative	2-arm parallel group RCT; Single-centre, open-label, coin-toss randomisation	*N* = 140 (IG = 70, CG = 70)	Schizophrenia; mean age IG = 26.27 ± 6.81, CG = 26.79 ± 6.99, male 54 (19%)	Psychosocial rehabilitation training + antipsychotic mono-medication/Receiving antipsychotic mono-medication	18 months	Positive and Negative Syndrome Scale (PANSS) Social Disability Screening Schedule (SDSS) Schizophrenia Cognition Rating Scale (SCRS)	Significant reduction in relapse rate at 18 months (*p* < 0.01), significant improvement in psychopathology, social functioning (both *p* < 0.05)	89%

*Abbreviations*: *ASI* Addiction Severity Index, *BAD* Bipolar Affective Disorder, *BPRS* Brief Psychiatric Rating Scale, *BSI* Brief Symptom Inventory, *CAU* Care as Usual, *CBR* Community-Based Rehabilitation, *CES* Coping Efficacy Scale, *CG* Control Group, *CGI-S* Clinical Global Impression-Severity Scale, *CIRE *Centre for Individual Rehabilitation and Education, *CM *Case Management, *CMP* Case-Managed Program, *CSES* Coping Self-Efficacy Scale, *DAS* Disability Assessment Schedule, *DISC-12* Discrimination and Stigma Scale, *GAF* Global Assessment of Functioning, *GAS* Goal Attainment Scaling, *HOPES* Helping Older People Experience Success, *HoNOS* Health of the Nation Outcome Scale, *IG* Intervention Group, *ILSS* Independent Living Skills Survey, *IMR* Illness Management and Recovery Program, *IMRS* Illness Management and Recovery Scale, *ISMI* Internalized Stigma of Mental Illness Scale, *Insight Assessment* Assessment of Insight, *Internalized Stigma* Internalized Stigma of Mental Illness Scale (ISMI), *MANS* Manchester Short Assessment of Quality of Life, *MARS* Maryland Assessment of Recovery in People with Serious Mental Illness, *MCAS *Multnomah Community Ability Scale, *MD *Major Depression, *MHRM* Mental Health Recovery Measure, *MMSE* Mini-Mental State Examination, *MSPSS* Multidimensional Scale of Perceived Social Support, *Medication Compliance* Medication Compliance Assessment, *N/A* Not Available, *OPC* Outpatient Care, *PAM* Patient Activation Measure, *PANSS* Positive and Negative Syndrome Scale, *PANSS-N* Positive and Negative Syndrome Scale for Schizophrenia - Negative Syndrome Subscale, *PECC* Psychosis Evaluation Tool for Common Use by Caregivers, *PHB* Personal Health Budget, *PRIME* PRogramme for Improving Mental health carE, *PSP *Personal and Social Performance Scale, *QLS* Quality of Life Scale, *RAS* Recovery Assessment Scale, *RCT* Randomized Controlled Trial, *RISE* Rehabilitation Intervention for Schizophrenia Empowerment, *RSES* Revised Self-Efficacy Scale, *S-ICM* Intensive Case Management Program, *SANS* Scale for the Assessment of Negative Symptoms, *SBS* Social Behaviour Schedule, *SDS* Social Disability Screening Schedule, *SERS-SF* Self-Esteem Rating Scale-Short Form, *SES* Self-Esteem Scale, *SF36* Social Function 36 Scale, *SHS* State Hope Scale, *SMCM* Strengths Model of Case Management, *SQLS* Schizophrenia Quality of Life Scale, *SWLS* Satisfaction with Life Scale, *Stigma Self-assessment* Stigma Self-assessment Scale, *Suicidality* Suicidality (assessed with a modified PECC suicidality subscale), *TAU* Treatment as Usual, *Treatment Adherence* Treatment Adherence, *UPSA* University of California at San Diego Performance-Based Skills Assessment, *VSSS-32 *Verona Service Satisfaction Schedule, *WAI* Working Alliance Inventory, *WCQ* Ways of Coping Questionnaire, *WHO* World Health Organization, *WHODAS *World Health Organization Disability Assessment Schedule, *WHOQOL-BREF* World Health Organization Quality of Life Scale

### Rehabilitation Model Types

The included studies were categorised as evaluating one of the following types of model, based on the theoretical approach and/or key components of the evaluated model.

#### Strengths-Based Models

There were four studies [27, 29, 32, 43] which examined case management models that used a strengths-based recovery approach. Unlike traditional deficit- or illness-focused approaches, these models employed an optimistic, goal-setting strategy that aimed to empower patients by emphasising their strengths and potential for growth and recovery and integrating community resources. Across all these studies, personal rather than clinical outcomes of interest were reported, including goal setting and/or attainment, quality of life, hope, empowerment, interpersonal relationships and self-efficacy, all of which were acknowledged as factors relevant to the recovery process in qualitative research on patients’ perspectives of these types of models [29].

#### Goal-Orientated Models

This group included two studies conducted in the Netherlands that investigated the Boston Psychiatric Rehabilitation Approach [41] and the Dutch model developed by the Centre for Individual Rehabilitation and Education [44]. These models assessed the attainment of personal rehabilitation goals in areas such as work, learning, and social contacts.

#### Holistic Care Models

Four studies [34–36, 42] evaluated holistic care models that adopted a multidisciplinary approach to deliver coordinated services providing medical, psychological, and social support. These models all aimed to enhance clinical recovery, personal recovery, and social functioning and as well as these domains, outcomes assessed included well-being, functional independence, stigma and discrimination, and social inclusion.

#### Community Rehabilitation Models in Low-Income Countries

Four studies reported on evaluations of the holistic care approach that had been culturally adapted for low-income countries - Ethiopia, South Africa, Indonesia and India [24–26, 37]. The model was implemented in community settings predominantly by lay or non-clinically trained staff, with the goal of reintegrating individuals into their communities while addressing social, cultural, and economic barriers. This model type was characterised by an emphasis on social inclusion, family involvement, and the utilisation of community resources, making them particularly relevant in settings with limited access to specialist mental health services.

#### Illness Management and Recovery Programmes

Five studies [28, 30, 33, 38, 39] reported on psychoeducation-based rehabilitation models, particularly the Illness Management and Recovery Programme. This approach, aimed at enhancing self-management and recovery for individuals with severe mental illness, is increasingly understood as a broader framework integrated into standard practice rather than as a standalone intervention. These studies assessed outcomes such as symptom reduction, improved functioning, and progress toward recovery goals.

#### Intensive Case Management Models

Two studies [31, 40] evaluated Intensive Case Management (ICM), an outreach model that provides individualised support to people with severe mental illness in their own homes by staff with relatively low caseloads. Delivered by multidisciplinary teams, studies of ICM primarily report outcomes such as reducing hospitalisations and improving psychosocial functioning.

#### Psychosocial Rehabilitation Models

Three studies [22, 23, 45] reported on psychosocial rehabilitation models that aimed to equip individuals with the emotional, cognitive, and social skills necessary for community living. Commonly reported outcomes of these studies were social functioning, quality of life, and psychopathology.

#### Similarities and differences between model types

Supplementary Tables 1 to 7 show how each of the included studies and the models they describe were allocated to one of the seven rehabilitation model types based on their key components. These tables also show the content, setting, staffing, and duration for each of the models described.

Although we were able to allocate each of the studies and the models they described as one of our rehabilitation model types based on their key defining features, there was substantial overlap between some of the model types. Out of the four studies allocated to the Strengths-Based model type, three of the studies described goal setting as an important part of the model. Goal setting was also an important feature of the Illness Management and Recovery Programme model type, where it was described as a component for each of the five included studies here. However, goal setting was not the defining feature of these models like it was for the models allocated to the Goal-Orientated models.

The models described in the studies allocated to the Holistic Care model type also had overlap with other model types. A biopsychosocial approach was described as a feature of three of the four models allocated to the Holistic Care model type, but was also a feature for three of the models allocated to the Community Rehabilitation in Low-Income Countries model type and both models allocated to the Intensive Case Management model type. However, what differentiated the models allocated to the Holistic Care model type from the other models was that a comprehensive approach to treatment was the defining feature of these models.

### Effectiveness of the Rehabilitation Models

#### Strengths-Based Models

While all four studies [27, 29, 32, 43] investigated models that share a common foundation in strengths-based approaches, they differed significantly in implementation strategies and the recovery outcomes they achieved. These differences were examined across different settings (three in community outpatient services and one in supported accommodation) and across multiple countries such as China [27], Australia [29], Israel [32] and Hong Kong [43], using diverse methodologies, including an RCT [32], a non-randomised controlled study [43], a longitudinal cohort study [27], and a qualitative study [29]. The mean Kmet quality score was high at 95%, with one study scoring 100% [43].

Although the interventions varied in content and delivery methods, all were centred around key personal recovery domains. The most consistent positive outcome across studies was improvement in goal setting and attainment. Gelkopf et al. [32] (quality score: 89%) observed significant improvements in goal attainment within the intervention group compared to the control group. Similarly, Tsoi et al. [43] (quality score: 100%) reported significant gains in the intervention group; however, caution is needed as the non-randomised design is likely to have introduced bias. Crucially, the only qualitative study of the Strengths-Based rehabilitation model—Dissanayake et al. [29] (quality score: 89%)—provides insight into how these models support goal setting and attainment, which emerged as a major theme and a key factor in recovery. Service-user interviews revealed three key elements: (i) a collaborative and hopeful relationship with the case manager; (ii) repeated use of the strengths assessment, which turns broad ambitions into specific, achievable steps; and (iii) a growing sense of personal agency that is sustained when goals are reviewed regularly—something that faded whenever Personal Recovery Plans (PRP), central tool in the model, were not revisited. Gelkopf et al. [32] also reported significant improvements in both quality of life and self-efficacy, along with a reduction in unmet needs, in their large trial, highlighting the effectiveness of their approach to Strengths-Based Case Management being incorporated within Psychiatric Rehabilitation Services (SBCM-PRS). In contrast, Tsoi et al. [43] and Cheng et al. [27] (quality score: 96%) did not observe significant improvements in these areas, but this could be due to their smaller sample sizes and shorter follow-up (12 months versus 20 months). Although all studies reported positive trends in personal recovery, none found significant differences in symptom severity. Authors cited various limitations that may have masked true effects, including missing adherence data, and the advanced illness stage of participants in Cheng et al. [27]. Gelkopf et al. [32] also noted baseline quality-of-life imbalances, use of locally developed outcome measures with limited validation, and variability in fidelity as possible contributors.

#### Goal-Orientated Models

The two studies in this category [41, 44] were both conducted in the Netherlands but varied in the settings and the outcomes assessed. Swildens et al.‘s [42] study employed a whole-system perspective, incorporating inpatient, outpatient, and sheltered living care, while van Busschbach et al. [44] focused on community outpatient services. One study was an RCT [41], and the other a prospective cohort study [44], both of high quality, with Kmet scores of 93% [41] and 95% [44].

Both studies examined models sharing a common foundation in the Boston Psychiatric Rehabilitation Approach and consistently reported positive outcomes in personal goal achievement and social participation. Swildens et al. [41] demonstrated a 21% higher rate of goal achievement, alongside a notable 21% increase in societal participation in the intervention group compared to controls. Van Busschbach et al. [44] found that 46% of participants fully achieved their goals, with significant reductions in unmet needs across social contacts, daily activities, and accommodation. However, the prospective design without a control group limits the ability to attribute these outcomes solely to the intervention. Additionally, neither study found significant improvements in quality of life or overall functioning, indicating that while effective for specific goals, the intervention may need additional components focusing on these. In addition, Swildens et al.‘s [41] study involved a population with a broad range of conditions, which may have diluted potential improvements in quality of life. In contrast, Van Busschbach et al. [44] had a smaller sample size (*n* = 35) and a shorter follow-up period (12 months), which may have left the study underpowered to detect meaningful change. The authors also did not use a formal fidelity tool and later acknowledged that staff training and oversight were unsystematic, raising uncertainty about adherence to the Boston model. This differs from Swildens et al. [41], who applied a 50-item psychiatric rehabilitation fidelity checklist and found that 86% of practitioners met “fair” or “good” standards.

#### Holistic Care Models

This group of studies [34–36, 42] varied the most in intervention focus and target populations, though all were based in community services. The studies were conducted in China [34, 42], the United States [35] and Italy [36]. All used quantitative methodologies, with two employing RCTs [34, 35], one a retrospective cohort study [36], and one a prospective non-randomised controlled study [42]. The mean Kmet quality score was 95%, with two studies achieving a score of 100% [36, 42].

All studies reported on models that employed a comprehensive (holistic) approach to community treatment, focusing on various aspects of rehabilitation. Regardless of the setting, whether community centres, mental health clinics, or home visits, the interventions consistently demonstrated positive outcomes in improving social functioning and reducing symptoms. For instance, Li et al. [34] (quality score: 86%) in China observed substantial reductions in psychopathology, improved social functioning, and reduced internalised stigma in their evaluation of an intervention that included cognitive behavioural therapy and psychoeducation, but no significant improvement in quality of life or medication compliance was noted. The authors suggested that the absence of family interventions, likely due to practical challenges, may have been a limiting factor in the intervention’s effectiveness. However, without objective fidelity data, it remains unclear whether this multicomponent package was implemented consistently across sites and sessions, potentially limiting the reliability of the reported outcomes. Mueser et al. [35] (quality score: 93%) studied a rehabilitation programme for older people with severe mental illness in the United States. With full fidelity to the intervention model (100% of 48 audited sessions rated at full adherence), the study found that men benefited more than women, showing improvements in social skills, psychosocial and community functioning—highlighting the need for further research into gender differences in response.

In Italy, Pelizza et al. [36] (quality score: 100%) demonstrated that the ‘Personal Health Budget’ model was associated with significant improvements in psychiatric symptoms, particularly negative symptoms, and improved both social and overall functioning, but these findings come from an uncontrolled cohort, so causal inference is very limited. Lastly, Tao et al. [42] (quality score: 100%) in China showed that the ‘Sunshine Heart Garden’ program, which integrated hospital and community services with a focus on societal reintegration and family education, was associated with significant improvements in psychopathology and social functioning, but there was no reduction in hospitalisation rates. The authors acknowledged that process-adherence data were not collected, and baseline imbalances further limit confidence in the findings. However, whilst all these studies demonstrated clinical and social improvements, personal recovery was not reported as an outcome.

#### Community Rehabilitation Models in Low-Income Countries

While all four studies [24–26, 37] in this category examined culturally adapted models that shared a common commitment to improving quality of life and social inclusion, they differed in their approach to the culture they were adapted to, implementation strategies and resources, and the recovery outcomes they reported. They were all conducted in community settings in low-income countries such as Ethiopia [24], South Africa [25], Indonesia [37] and India [26]. The studies employed diverse methodologies, including RCT [24], a quasi-experimental study [37], a prospective cohort study [26] and a qualitative study [25]. The mean Kmet quality score was 98%, with three studies achieving the maximum score of 100% [24, 26, 37].

Chatterjee et al. [26] (quality score: 100%) in India evaluated a culturally adapted model of support for people with long-term schizophrenia that combined psychiatry with traditional community support, involving mental health workers, families, and traditional healers. The approach was associated with improved psychopathology and reduced disability, but caution in the interpretation of results is warranted due to missing follow-up data and the absence of any fidelity assessment. Asher et al. [24] (quality score: 100%) in Ethiopia used an approach that combined home- and office-based rehabilitation support from non-specialist workers. Ninety-four per cent of participants completed every core session (high fidelity), and at 12 months, the programme produced significant reductions in disability and symptom severity. Puspitosari et al. [37] (quality score: 100%), in Indonesia, evaluated community-based delivery of rehabilitative support delivered by primary care physicians and community mental health nurses and found a significant increase in quality of life in the intervention group compared to the control group, yet follow-up ended at just 16 weeks. Brooke-Sumner et al. [25] (quality score: 90%), the only qualitative study in this group, conducted on a community-based model in South Africa delivered by local health workers, offers explanatory depth that helps connect these quantitative gains to lived experiences. Service-user interviews identified three pathways through which such models facilitated change: (i) enhanced personal empowerment (e.g. increased self-esteem, renewed hope, stronger sense of agency), (ii) increased social connectedness (e.g. reduced isolation, improved family relationships, new peer friendships), and (iii) practical life-role gains (e.g. improved medication adherence, budgeting, resumption of household tasks, and re-engagement with community life). While the variations in intervention content and delivery mode of these models make comparison of their effectiveness challenging, they highlight the adaptability of these models across different contexts.

#### Illness Management and Recovery Programmes

The interventions that were evaluated in these studies [28, 30, 33, 38, 39] all focused on recovery promotion through structured models based on the Illness Management and Recovery (IMR) programme but differed in their implementation strategies, integration methods and the recovery outcomes reported. These studies were all community-based (three in community outpatient services, one in supported accommodation) and conducted in Sweden [30], Israel [33], the United States [39], the Netherlands [38], and Denmark [28]. All used RCT design, and the average Kmet quality score was 89%.

Färdig et al. [30] (quality score: 89%) and Roosenschoon et al. [38] (quality score: 93%) reported significant improvements in self-management, symptoms, and coping strategies, though neither study found the intervention to be associated with gains in quality of life or hospitalisation rates compared to usual care. The authors themselves explained the null findings as follows: Färdig et al. [30] pointed to their small 41-person sample and baseline ceiling/floor effects—participants already had high quality-of-life and recovery scores and few recent hospitalisations, leaving little room for further improvement, whereas Roosenschoon et al. [38] attributed the lack of impact to only half the patients completing 50% or more of the sessions, fair-to-moderate IMR fidelity, and an already high standard of Dutch usual care that limited added value. Hasson-Ohayon et al. [33] (quality score = 82%) likewise found improvements in psychopathology and coping, but no gain in social support. Fidelity was good (mean IMR score: 3.9/5), and the authors suggested the null effect may reflect the greater behavioural complexity required to build social networks compared to acquiring illness knowledge. In contrast, Salyers et al. [39] (quality score: 93%) and Dalum et al. [28] (quality score: 89%) found no significant improvements in illness management or personal recovery, despite Dalum et al.’s [28] integration of IMR with treatment as usual (TAU), which was expected to enhance the intervention’s effectiveness. This may reflect sub-therapeutic exposure—only 28% of participants in Salyers et al. [39] attended over half of the sessions, and Dalum et al. [28] reported a mean of 16/53 sessions were attended, with around half of the participants attending less than half of the sessions. In addition, both studies reported only moderate fidelity to the IMR programme. Färdig et al. [30], Hasson-Ohayon et al. [33], and Roosenschoon et al. [38] all showed more positive outcomes when family and peer support were incorporated into the intervention.

#### Intensive Case Management Models

This group comprised two studies that varied significantly in intervention implementation and the breadth of outcomes measured. Both focused on community outpatient services. One was a retrospective cohort study [40] conducted in Korea, and the other was a prospective case-control study [31] from Spain. Both were of high quality, with Kmet scores of 95% [31] and 100% [40], respectively.

Both studies evaluated intensive case management delivered in community settings, and both demonstrated the intervention to be associated with reduced inpatient service use. Fernandez-Miranda et al. [31] (quality score: 95%) reported a significant reduction in hospitalisations, with 17.4% of patients in the intervention group being hospitalised compared to 46.5% in the control group (and an average of 0.9 hospitalisations versus 3.2) over ten years. Sohn et al. [40] (quality score: 100%) reported a reduction in the average length of hospital stay from 1.47 pre-intervention to 0.26 days per month post-intervention, with sustained effects. Additionally, Fernandez-Miranda et al. [31] reported that the intervention group showed greater improvements in psychopathology, fewer suicide attempts, and better treatment adherence than the control group, though this may be partially explained by the fact that more of those in the intervention group receive long-acting injectable antipsychotic medication. Nevertheless, the findings from these two studies concur with many earlier studies that have found ICM to be associated with reduced inpatient service use.

#### Psychosocial Rehabilitation Models

This group of studies varied significantly in settings, intervention content, and the breadth of recovery outcomes. One study was conducted in an inpatient setting in Sudan [22], while the other two were based in community outpatient services in Turkey [23] and China [45]. The study designs included a quasi-experimental design [22], a controlled clinical study [23], and an RCT [45], all of high quality, with Kmet scores ranging from 89% [45] to 95% [22, 23].

All three studies used a psychosocial rehabilitation approach and consistently reported significant improvements in psychopathology and social functioning among participants with schizophrenia. Ahmed et al. [22] (quality score: 95%) focused on enhancing social and cognitive functioning, reporting significant improvements in these areas as well as in psychopathology. However, the generalisability of their findings is limited by the smaller, male-only sample (n = 49). In contrast, Arslan et al. [23] (quality score: 95%), with a larger and more diverse sample (n = 104), observed improvements for those receiving intervention not only in psychopathology and social functioning but also in quality of life and insight. The intervention was built around therapeutic relationships, stigma reduction, and personalised rehabilitation goals, with a stronger focus on personal recovery. Complementing these findings, Wang et al. [45] (quality score: 89%) reported reduced relapse rates, which might be attributed to the combined use of psychosocial rehabilitation and pharmacological treatment. In this study, relapse was defined as the recurrence of severe psychotic symptoms (based on PANSS score criteria), significant functional disturbances (e.g., self-harm, harm to others), or the need for rehospitalisation. However, caution is warranted, as none of the three studies reported any structured fidelity monitoring.

### Consistencies and differences between model types in effectiveness

Although the models differed in their theoretical underpinnings and targeted outcomes, they all had a positive impact on people with complex psychosis. Strengths-Based and Goal-Oriented Models most consistently improved personal recovery, while Intensive Case Management Models stood out for reducing hospital use. Holistic Care Models and Community Rehabilitation Models in Low-Income Countries, as well as Psychosocial Rehabilitation Models, were consistently associated with improvements in symptoms and social functioning, with the latter also showing improvements in quality of life. The evidence for Illness Management and Recovery Models were less consistent but studies did often report improvements in self-management and coping.

Most models were delivered in community or supported accommodation settings, with limited use in inpatient environments. A quarter of the included studies (six out of 24) emphasised the importance of integration with other local services or the community [24, 26, 32, 36, 37, 42]. They consistently framed integration—via case-managers embedded in multi-agency networks [32], hospital-to-community pathways [24, 26, 37, 42] and pooled health-social care budgets [37]—as the mechanism that secured employment, housing and financial supports, boosted treatment engagement and medication adherence, and substantially reduced disability or rehospitalisation compared with stand-alone clinic care. 

## Discussion

This systematic review is the first to compare international mental health rehabilitation models for individuals with complex psychosis. A range of models were identified and grouped into seven categories, based on similarities in focus and approach. The 24 studies identified for inclusion in the review were too heterogeneous for meta-analysis, so a narrative synthesis was conducted. Heterogeneity was due to variation in the content of the interventions, study design and outcomes reported. While most of the models were shown to be effective in areas such as clinical, personal and social recovery, no single approach was found to be universally superior.

Many of the evaluations reported positive outcomes for models that operated within a recovery-oriented culture that facilitated individualised, person-centred care. This aligns with findings from large national research programmes in the UK, which show that recovery-based practice is associated with improved outcomes in mental health rehabilitation and supports individuals' progress toward independent living [9, 10]. Strengths-Based and Goal-Oriented Models, in particular, embodied these principles and were associated with improvements in goal setting and attainment, although their impact on clinical symptoms and social functioning was less evident. These models typically involved individual, community-based sessions every 2–3 weeks, mostly delivered by trained case managers, and generally lasted around 12 months. Models that offered a range of biopsychosocial interventions delivered by a multidisciplinary team were associated with better clinical outcomes in several evaluations, reflecting the recommendations of the NICE Guideline on Rehabilitation for Adults with Complex Psychosis [3]. These models offered more frequent contact, such as weekly individual or group sessions, and lasted around 24 months. Holistic Care Models, in particular, demonstrated positive outcomes in reducing clinical symptoms and improving social functioning. Intensive Case Management Models further showed reduced hospitalisation and improved treatment adherence, likely due to their emphasis on smaller caseloads, personalised outreach, and high-intensity support—reflecting Harvey et al.’s [18] recognition of ICM as a well-established approach to supporting clinical recovery. However, the lack of recovery-oriented outcome measures in ICM evaluations limits understanding of their broader impact on personal recovery.

Psychosocial Rehabilitation Models, which focused particularly on cognitive and social impairments, were consistently linked with improvements not only in psychopathology but also in cognitive and social functioning, as well as overall quality of life. These models, alongside Illness Management and Recovery programmes, frequently integrated core components such as structured psychoeducation and social skills training—both of which are manualised, scalable, and can be delivered by trained staff across settings. Family and peer involvement was also notable within these models and in Illness Management and Recovery programmes, where it was associated with improved self-management, symptom reduction, and coping skills.

These findings align with the principles of the Dutch ‘Active Recovery Triad’ (ART) model for people with severe mental illness [13], which emphasises collaboration between service users, families, and professionals. Collaboration also extended into the wider community, as seen in Holistic Care Models, Strengths-Based Models, and Community Rehabilitation Models in Low-Income Countries, which demonstrated how community integration and local connections can help build strong support systems that facilitate recovery and social integration.

Contextual adaptation was further illustrated by models developed for resource-constrained settings. Community Rehabilitation Models in Low-Income Countries, for instance, were delivered by trained lay or non-specialist staff and showed positive outcomes across multiple domains—including reductions in psychopathology, enhanced quality of life, increased social support, and reduced social isolation. A similar approach proved effective in a region with unique geographic and resource limitations in Tasmania, where Savaglio et al. [46] found that a community-based recovery program improved psychosocial functioning in people with complex mental health needs. Model variation and flexibility according to local structure and need is inevitable and should be preferred to a fixed uniform approach to rehabilitation. However, understanding the core components of effective rehabilitation models and incorporating these components in models where they are absent and where it is appropriate to do so, should improve individual outcomes.

Finally, it is recommended by NICE [3] that a local pathway approach should be taken to rehabilitation. The pathway should include different types of services that can provide different types and levels of support, such as specialist inpatient rehabilitation units, community rehabilitation teams, and supported accommodation. This pathway should be highly integrated and connected to local resources including employment support, education, and leisure. Integration with other rehabilitation services and connection to the local community was largely missing from the models described in the studies included in this review. Only six out of the 24 [24, 26, 32, 36, 37, 42] included studies mentioned integration with other services. However, it is an important aspect of rehabilitation services and the long-term recovery of people with complex psychosis.

### Strengths and limitations

The review included an extensive search of the international literature across six databases, yielding 6,939 articles post-deduplication, supplemented by forward and backward citation searches. Decisions about including individual studies were validated by a second researcher screening a sample of the studies at both stages of the search, and by double rating a sample of the included studies for quality. The inclusion of diverse study designs and settings strengthens the generalisability of the findings. Finally, the review was prospectively registered, reducing potential bias in the conduct and reporting of results.

The main limitation of this review was the heterogeneity of the included studies, which covered a broad range of rehabilitation models from different countries that varied in reported outcomes and design, making them too diverse for meta-analysis. Additionally, the term 'mental health rehabilitation model' encompasses a wide range of approaches internationally, and the lack of clear distinctions between complex rehabilitation models and other supportive services or treatment programs made it challenging to consistently categorise interventions, potentially leading to the inadvertent exclusion of relevant studies. It is also possible that a model that meets our criteria for a mental health rehabilitation model but is not described as a ‘rehabilitation model’ or another term that we have used in our search strategy for this concept (see Additional file 1: Final Search Strategy) in the published article and would therefore not be included in our searches. Indeed, this is the case for a study by Dabholkar et al. [47], which evaluated a model that integrated a recovery-based community service with tertiary care hospitals that was designed to support people with schizophrenia in low- and middle-income countries. If included in our review, this study would have been included in our Community Rehabilitation Models in Low-Income Countries model type. The study was an 18-month cohort study with no comparison group, and a sample size of 239 individuals. They reported positive outcomes, including a reduction in disability (as measured by the Indian Disability Evaluation and Assessment Scale [48] and in the number of unmet needs (as measured by the Adapted Camberwell Assessment of Needs Scale [49]. Primary caregivers also reported fewer difficulties relating to unemployment, interpersonal conflicts, and social isolation. This study, in addition to the four studies we included in our Community Rehabilitation Models in Low-Income Countries model type, adds further strength to the feasibility of implementing recovery-based community rehabilitation in low- and middle-income countries.

Another limitation of our review relates to our eligibility criteria for the population or sample of the study was broad and may have included studies of adults which did not meet NICE’s definition of complex psychosis. We included studies where at least half of the sample had a primary diagnosis of schizophrenia, schizoaffective disorder, bipolar affective disorder, or severe depression with psychosis. We excluded studies of models which specifically targeted first episode psychosis or children and adolescents with psychosis. We did not include eligibility criteria relating to what makes psychosis ‘complex’, such as indicators of treatment resistance, co-existing conditions, or difficulties with social and everyday functioning. Adding this to our eligibility criteria would have increased our confidence that the samples of he included studies did meet NICE’s definition of complex psychosis. However, it would have also excluded many studies of mental health rehabilitation which did not report these characteristics of their sample but are still of relevance to this review and to people with complex psychosis. Finally, the exclusion of grey literature and non-English language studies means that some relevant papers may not have been identified for potential inclusion.

### Future implications

This systematic review identified seven distinct models of mental health rehabilitation for people with complex psychosis. Whilst this evidence of interest in this field is encouraging, greater synergy is needed to consolidate these into a more universally accepted and specified model that incorporates the core components that this review has identified as most beneficial. Such a model should incorporate evidence-based biopsychosocial interventions, including family and peer support, aim to improve individual’s social and everyday living skills, and optimise their community integration and quality of life. It should encompass a recovery-based approach that builds on the individual’s strengths and helps them to identify and work towards specific, personalised goals, collaboratively. Such person-centred and recovery-oriented practice is already recommended by NICE [3] for this client group to facilitate clinical, personal, and social recovery. However, further research is needed to ensure that such a model incorporates service users’ perspectives to ensure it has the flexibility to be responsive to their needs and preferences. Longer-term studies are also needed to understand whether the gains associated with this kind of comprehensive rehabilitative model can be sustained when the intervention reduces in intensity, and to assess its adaptability to different socioeconomic and cultural contexts.

## Conclusion

A range of mental health rehabilitation models exists globally. Whilst some models appear suited to certain contexts and some have demonstrated effectiveness in regard to specific outcomes, a more universal, biopsychosocial rehabilitative approach to the treatment and support of people with complex psychosis is needed, incorporating the specific ‘core components’ identified in this review. However, a degree of flexibility is required to ensure the model can be effectively implemented in local settings.

## Supplementary Information

Additional file 1. Final Search Strategy. The search strategy used for the systematic review, organised according to the databases

Additional file 2. Supplementary Tables 1–7. Study Allocation by Rehabilitation Model Type and Key Components

## Data Availability

All data supporting the findings of this systematic review are included in the manuscript. The full search strategy and supplementary Tables 1-7 are provided in the Supplementary Material. No additional datasets were generated or analysed beyond those reported in the included studies.

## Abbreviations

ACT: Assertive Community Treatment
ADL/ADLs: Activities of Daily Living
ART: Active Recovery Triad
ASI: Addiction Severity Index
BAD: Bipolar Affective Disorder
BPRS: Brief Psychiatric Rating Scale
BSI: Brief Symptom Inventory
CAU: Care as Usual
CBR: Community-Based Rehabilitation
CBR-LIC: Community-Based Rehabilitation in Low-Income Countries
CBT: Cognitive Behavioural Therapy
CES: Coping Efficacy Scale
CG: Control Group
CGI-S: Clinical Global Impression-Severity Scale
CIRE: Centre for Individual Rehabilitation and Education
CM: Case Management
CMHC: Community Mental Health Centre
CMP: Case-Managed Programme
CSES: Coping Self-Efficacy Scale
DAS: Disability Assessment Schedule
DISC-12: Discrimination and Stigma Scale
GAF: Global Assessment of Functioning
GAS: Goal Attainment Scaling
HOPES: Helping Older People Experience Success
HoNOS: Health of the Nation Outcome Scale
ICM: Intensive Case Management
IG: Intervention Group
ILSS: Independent Living Skills Survey
IMR: Illness Management and Recovery
IMRS: Illness Management and Recovery Scale
Insight Assessment: Assessment of Insight
Internalized Stigma: Internalized Stigma of Mental Illness Scale (ISMI)
IRM: Integrated Recovery-oriented Model
IRP: Individual Rehabilitation Plan
ISMI: Internalized Stigma of Mental Illness Scale
LAIs: Long-Acting Injectables
MANS: Manchester Short Assessment of Quality of Life
MARS: Maryland Assessment of Recovery in People with Serious Mental Illness
MCAS: Multnomah Community Ability Scale
MD: Major Depression
MHRM: Mental Health Recovery Measure
MMSE: Mini-Mental State Examination
MSPSS: Multidimensional Scale of Perceived Social Support
N/A: Not Available
NHS: National Health Service
NGO: Non-Governmental Organisation
NICE: National Institute for Health and Care Excellence
n: sample size/number of participants
OPC: Outpatient Care
OR: Odds Ratio
PAM: Patient Activation Measure
PANSS: Positive and Negative Syndrome Scale
PANSS-N: PANSS – Negative Syndrome Subscale
PECC: Psychosis Evaluation Tool for Common Use by Caregivers
PHB: Personal Health Budget
PR: Psychiatric Rehabilitation/Psychosocial Rehabilitation
PRIME: PRogramme for Improving Mental health carE
PRP: Personal Recovery Plan
PSP: Personal and Social Performance Scale
PsRT: Psychosocial Rehabilitation Training
QLS: Quality of Life Scale
RAS: Recovery Assessment Scale
RCT: Randomized Controlled Trial
RISE: Rehabilitation Intervention for Schizophrenia Empowerment
RSES: Revised Self-Efficacy Scale
S-ICM: Seoul-Intensive Case Management
SANS: Scale for the Assessment of Negative Symptoms
SASD: Strategies Against Stigma and Discrimination
SBCM: Strengths-Based Case Management
SBCM-PRS: Strengths-Based Case Management + Psychiatric Rehabilitation Services
SBS: Social Behaviour Schedule
SDS: Social Disability Screening Schedule
SERS-SF: Self-Esteem Rating Scale – Short Form
SES: Self-Esteem Scale
SF36: Social Function 36 Scale
SHS: State Hope Scale
SMCM: Strengths Model of Case Management
SQLS: Schizophrenia Quality of Life Scale
Stigma Self-assessment: Stigma Self-assessment Scale
Suicidality: Assessed with a modified PECC suicidality subscale
TAU: Treatment as Usual
Treatment Adherence: Treatment Adherence
UPSA: University of California at San Diego Performance-Based Skills Assessment
VSSS-32: Verona Service Satisfaction Schedule
WAI: Working Alliance Inventory
WCQ: Ways of Coping Questionnaire
WHO: World Health Organization
WHODAS: World Health Organization Disability Assessment Schedule
WHOQOL-BREF: World Health Organization Quality of Life Scale
