# Supplementary material for: Mental health rehabilitation models for people with complex psychosis: a systematic review
Source: BMC Psychiatry. 2025 Aug 26;25:812. doi: 10.1186/s12888-025-07174-8 (PMC12379344; doi:10.1186/s12888-025-07174-8)
Supplement: Supplementary file 1 — Additional file 1. Final Search Strategy. The search strategy used for the systematic review, organised according to the databases [file 12888_2025_7174_MOESM1_ESM.docx]

**Supplementary material**

**Final Search Strategy**

| **MEDLINE (OVID)** | | **EMBASE (OVID)** | | **APA PsycINFO (OVID)** | |
| --- | --- | --- | --- | --- | --- |
| *N* | *Terms* | *N* | *Terms* | *N* | *Terms* |
| 1 | exp Psychotic Disorders/ or Bipolar Disorder/ or Schizophrenia/ | 1 | exp Psychotic Disorders/ or Bipolar Disorder/ or Schizophrenia/ | 1 | exp Psychotic Disorders/ or Bipolar Disorder/ or Schizophrenia/ |
| 2 | (Schizophren* OR Schizoaffective disorder* or Bipolar affective disorder* or Psychotic depression or Chronic psychosis or Delusional disorder*).ti,ab. | 2 | (Schizophren* or Schizoaffective disorder* or Bipolar affective disorder* or Psychotic depression or Chronic psychosis or Delusional disorder*).ti,ab. | 2 | (Schizophren* or Schizoaffective disorder* or Bipolar affective disorder* or Psychotic depression or Chronic psychosis or Delusional disorder*).ti,ab. |
| 3 | ((serious or severe) adj3 ("mental illness*" or "mental disorder*")).ti,ab. | 3 | ((serious or severe) adj3 ("mental illness*" or "mental disorder*")).ti,ab. | 3 | ((serious or severe) adj3 ("mental illness*" or "mental disorder*")).ti,ab. |
| *4* | *1 or 2 or 3* | *4* | *1 or 2 or 3* | *4* | *1 or 2 or 3* |
| 5 | ((care or recovery or rehabilitation) adj5 (model* or plan* or methodolog* or pathway*)).ti,ab. | 5 | ((care or recovery or rehabilitation) adj5 (model* or plan* or methodolog* or pathway*)).ti,ab. | 5 | ((care or recovery or rehabilitation) adj5 (model* or plan* or methodolog* or pathway*)).ti,ab. |
| 6 | "Case management".ti,ab. | 6 | "Case management".ti,ab. | 6 | "Case management".ti,ab. |
| 7 | *5 or 6* | 7 | *5 or 6* | 7 | *5 or 6* |
| 8 | *4 and 7* | 8 | *4 and 7* | 8 | *4 and 7* |
| 9 | limit 8 to (english language and humans and yr="2000 -Current") | 9 | limit 8 to (english language and humans and yr="2000 -Current") | 9 | limit 8 to (english language and humans and yr="2000 -Current") |
|  |  |  |  |  |  |
| **Emcare (OVID)** | | **CINAHL Plus (EBSCO)** | | **Cochrane Library** | |
| *N* | *Terms* | *N* | *Terms* | *N* | *Terms* |
| 1 | exp Psychotic Disorders/ or Bipolar Disorder/ or Schizophrenia/ | S1 | (MH "Psychotic Disorders") OR (MH "Bipolar Disorder") OR (MH "Schizophrenia") | #1 | Psychotic Disorders OR Bipolar Disorder OR Schizophrenia |
| 2 | (Schizophren* or Schizoaffective disorder* or Bipolar affective disorder* or Psychotic depression or Chronic psychosis or Delusional disorder*).ti,ab. | S2 | (Schizophren* OR "Schizoaffective disorder*" OR "Bipolar affective disorder*" OR "Psychotic depression" OR "Chronic psychosis" OR "Delusional disorder*") | #2 | Schizophren* OR Schizoaffective disorder* OR Bipolar affective disorder* OR Psychotic depression OR Chronic psychosis OR Delusional disorder* |
| 3 | ((serious or severe) adj3 ("mental illness*" or "mental disorder*")).ti,ab. | S3 | ((Serious OR Severe) ADJ3 ("Mental illness*" OR "Mental disorder*")) | #3 | ((serious OR severe) NEAR/3 ("mental illness" OR "mental disorder")) |
| *4* | *1 or 2 or 3* | S4 | *S1 OR S2 OR S3* | #4 | *#1 OR #2 OR #3* |
| 5 | ((care or recovery or rehabilitation) adj5 (model* or plan* or methodolog* or pathway*)).ti,ab. | S5 | ((Care OR Recovery OR Rehabilitation) ADJ5 (Model* OR Plan* OR Methodolog* OR Pathway*)) | #5 | ((care OR recovery OR rehabilitation) NEAR/5 (model* OR plan* OR methodolog* OR pathway*)) |
| 6 | "Case management".ti,ab. | S6 | "Case management" | #6 | "Case management" |
| 7 | *5 or 6* | S7 | *S5 OR S6* | #7 | *#5 OR #6* |
| 8 | *4 and 7* | S8 | *S4 AND S7* | #8 | *#4 AND #7*  with Cochrane Library publication date Between Jan 2000 and May 2024 (Word variations have been searched) |
| 9 | limit 8 to (english language and humans and yr="2000 -Current") | S9 | S8 (Limiters – Publication year: 2000-2024; human; narrow by language: - english) |  |  |
