# Supplementary material for: Mental health rehabilitation models for people with complex psychosis: a systematic review
Source: BMC Psychiatry. 2025 Aug 26;25:812. doi: 10.1186/s12888-025-07174-8 (PMC12379344; doi:10.1186/s12888-025-07174-8)
Supplement: Supplementary file 2 — Additional file 2. Supplementary Tables 1–7. Study Allocation by Rehabilitation Model Type and Key Components [file 12888_2025_7174_MOESM2_ESM.pdf]

Mental Health Rehabilitation Models for People with Complex Psychosis: A Systematic Review

Nikola Houngbedji, Christian Dalton-Locke, Amelia Hughes, Helen Killaspy

BMC Psychiatry, 2025

Supplementary materials

**Supplementary Table 1: Strengths-Based Models**						
**First author and publication year**	**Name of study model**	**Key components**	**Content of model**	**Setting and country**	**Staffing**	**Duration**
Cheng et al. 2020 (27)	Strengths-Based Case Management (SBCM)	- Strengths-based (focused on clients' abilities, resources, and potential)<br>- Case management (individualised planning, coordination, ongoing monitoring)<br>- Structured psychoeducation (clients & family)	Comprehensive strengths assessment (mental & physical health, stress coping, social interactions, work/education/leisure, daily living skills, financial management, family support); individualised recovery plans; weekly psychiatrist medication reviews; weekly psychoeducation groups (clients & families); emergency intervention; regular contacts via face-to-face/phone; fortnightly contact initially, then monthly	Community outpatient services, China	Case managers (experienced nurses, doctors, social workers; receive pre-service training, booster sessions, weekly multidisciplinary supervision) with a maximum caseload of 15	12 months
Dissanayake et al. 2024 (29)	Strengths Model of Case Management (SMCM)	- Strengths-based<br>- Recovery-based<br>- Goal setting (client-led, collaboratively identified)<br>- Case management	Guided by the six SMCM principles- hope, strengths focus, primacy of the worker-client relationship, consumer-driven, use of community resources, community setting; eight-domain strengths assessment → co-created personal recovery plan (PRP) and goals; ongoing one-to-one strengths conversations; advocacy / linkage to community resources	Community outpatient services, Australia	Case managers	Not reported

**Supplementary Table 1: Strengths-Based Models (Continued)**						
**First author and publication year**	**Name of study model**	**Key components**	**Content of model**	**Setting and country**	**Staffing**	**Duration**
Gelkopf et al. 2016 (32)	Strengths-Based Case Management (SBCM) integrated with psychiatric rehabilitation services (PRS)	- Strengths-based<br>- Recovery-based<br>- Goal setting (client-led, collaboratively identified)<br>- Case management	Individualised recovery plans & goal setting; ongoing advocacy and liaison with PRS providers (education, employment, housing, family); 10 face-to-face or telephone contacts plus crisis response as needed	Community outpatient services, Israel	Case managers (60-hour SBCM training, weekly supervision) with a maximum caseload of 32	20 months
Tsoi et al. 2019 (43)	Strengths Model of Case Management (SMCM)	- Strengths-based<br>- Recovery-based<br>- Goal setting (client-led, collaboratively identified)<br>- Case management	Comprehensive strengths assessment; individualised recovery plan & goal setting; progress review; face-to-face individual sessions every 2–3 weeks (30–60 minutes), preferably in community settings e.g., parks or fast-food locations	Supported accommodation, Hong Kong	Caseworkers (2-days SMCM training, bi-monthly group supervision via Skype); caseload not reported	12 months

Abbreviations: SBCM — Strengths-Based Case Management; SMCM — Strengths Model of Case Management; PRS — Psychiatric Rehabilitation Services; PRP — Personal Recovery Plan

**Supplementary Table 2: Goal-Orientated Models**						
**First author and publication year**	**Name of study model**	**Key components**	**Content of model**	**Setting and country**	**Staffing**	**Duration**
Swildens et al. 2011 (41)	Boston Psychiatric Rehabilitation Approach (PR)	- Goal-orientated<br>- Recovery-based<br>- Skill-based<br>- Support person involvement	Three-phase cycle (Choose → Get → Keep): setting a self-defined goal in work, education, social life, or living situation; planning the required skills, supports, and resources with a professional caregiver; carrying out the interventions; individual sessions occur at least once every three weeks	Inpatient care, outpatient care and/or sheltered living, Netherlands	Mental health professionals (social workers, nurses, vocational therapists; training, monthly supervision)	24 months
Van Busschbach et al. 2002 (44)	Centre for Individual Rehabilitation and Education (CIRE)-based on the Boston University Model	- Goal-orientated<br>- Recovery-based<br>- Skill-based<br>- Support person involvement	Three-phase cycle (Diagnostic → Planning → Intervention): assessing strengths and readiness; setting a rehabilitation goal and plan; providing skills training and resource brokering for housing, work, education, or social network; flexible, client-determined sessions	Community outpatient services, Netherlands	Eight part-time counsellors (training, supervision)	12 months

Abbreviations: PR — Psychiatric Rehabilitation; CIRE — Centre for Individual Rehabilitation and Education

Supplementary Table 3: Holistic Care Models						
**First author and publication year**	**Name of study model**	**Key components**	**Content of model**	**Setting and country**	**Staffing**	**Duration**
Mueser et al. 2010 (35)	Helping Older People Experience Success (HOPES) programme	- Comprehensive, multicomponent<br>- Recovery-based<br>- Integrated (social rehabilitation + physical-health care)<br>- Biopsychosocial approach<br>- Skill-based (inc. in-vivo community-based practice)\<br>- Structured family involvement<br>- Multidisciplinary delivery	Weekly social-skills classes (90 min AM + 60 min PM, class size 6–8); twice-monthly community practice outings; monthly one-to-one nurse sessions (physical-health goals); workbook homework and family involvement when available. Two-year programme: year 1 intensive (full schedule), year 2 maintenance<br><br>+ TAU (pharmacotherapy, case management or outreach by non-users, individual therapy, and rehabilitation services, such as groups and psychoeducation)	Community outpatient services, United States	Nurses, social workers, occupational therapists, peer specialists (2-day training, monthly supervision)	24 months
Li et al. 2018 (34)	Community-based comprehensive intervention	- Comprehensive, multicomponent<br>- Recovery-based<br>- Biopsychosocial approach<br>- Skill-based<br>- Multidisciplinary delivery	24 modules delivered in eight phases: strategies against stigma & discrimination (SASD): stigma background, rights in employment/ education/ matrimony, coping skills; psychoeducation; social skills training; cognitive behavioural therapy; medication; monthly sessions (first 6 months), twice in final 3 months; 3×120-min modules per phase	Community outpatient services, China	Experienced psychiatrists, one psychotherapist, one social worker (1-day intensive training, supervision)	9 months

Supplementary Table 3: Holistic Care Models (Continued)						
**First author and publication year**	**Name of study model**	**Key components**	**Content of model**	**Setting and country**	**Staffing**	**Duration**
Tao et al. 2012 (42)	Sunshine Heart Garden programme	- Comprehensive, multicomponent<br>- Recovery-based<br>- Integrated (hospital + community services)<br>- Biopsychosocial approach<br>- Skill-based (in-vivo community-based practice)<br>- Structured family involvement<br>- Multidisciplinary delivery	Emergency response (timely intervention); rapid referral; rehabilitation services (Liberman-based training in medication self-management, symptom monitoring, social skills, recreational activities, physical exercise); family education and support (manual + 6 monthly 80–90 min sessions); trial employment and community reintegration; daily attendance; skills classes once or twice per month	Community outpatient services, China	Community health centre physicians, therapists from specialist hospitals, and community mental health service workers (pre-service training, supervision not reported)	12 months
Pelizza et al. 2023 (36)	Personal Health Budget (PHB)	- Comprehensive, multicomponent<br>- Recovery-based<br>- Integrated (social rehabilitation + health-care model)<br>- Co-produced Individual Rehabilitation Plan (IRP) (clients+ family + third sector)<br>- Multidisciplinary delivery	Individualised Rehabilitation Plans (IRPs) (housing, sociality, employment); co-developed with clients, families and local community agencies; IRP review every month	Community outpatient services, Italy	Mental-health clinicians, municipal social-service staff; family members and third-sector cooperatives	24 months

Abbreviations: HOPES — Helping Older People Experience Success; TAU — Treatment as Usual; SASD — Strategies Against Stigma and Discrimination; CBT — Cognitive Behavioural Therapy; IRP — Individual Rehabilitation Plan; PHB — Personal Health Budget

Supplementary Table 4: Community Rehabilitation Models in Low-Income Countries						
**First author and publication year**	**Name of study model**	**Key components**	**Content of model**	**Setting and country**	**Staffing**	**Duration**
Asher et al. 2022 (24)	Community-Based Rehabilitation (CBR) + task-shared facility care (RISE trial)	- Comprehensive, multicomponent<br>- Recovery-based<br>- Rights-based & stigma-reduction focus<br>- Integrated (home-visit rehab + primary-care medication management)<br>- Structured family involvement<br>- Multidisciplinary, task-shared delivery<br>- Culturally adapted (local beliefs, translated content, lay worker delivery)	Home-visit package (30–90 min) over three phases: 1) engagement, 2) individual goals, 3) sustaining progress. Core tasks: psychoeducation, adherence support, family intervention, crisis management, coping with stigma, return-to-work coaching. CBR workers also mobilise community resources, run family-support groups and strengthen links with facility care. Facility-based care supplies antipsychotics and brief psychoeducation.	Community outpatient services, Ethiopia	11 lay CBR workers (≥10 years of education, no mental health background); 5-week (150 h) training in CBR delivery, basic counselling, and problem-solving (delivered by psychiatrists and CBR coordinators); monthly half-day refresher sessions; ongoing supervision by 2 supervisors	12 months
Brooke-Sumner et al. 2017 (25)	Community-based Psychosocial Rehabilitation (CBR)	- Comprehensive, multicomponent<br>- Recovery-based<br>- Manualised<br>- Skill-based<br>- Goal setting<br>- Structured psychoeducation<br>- Structured family involvement<br>- Task-shared delivery<br>- Culturally adapted (local beliefs, translated content, lay worker delivery)	12 weekly manual-based sessions (60-90 min) for clients: goal setting and psychoeducational sessions on schizophrenia, covering diagnosis, medication, substance abuse, coping strategies, insight, stress, stigma management and practical life skills, homework + 5 sessions for caregivers	Community outpatient services, South Africa	Two non-specialist auxiliary social workers; 5-day training + facilitator guide; supervision (Setswana-speaking supervisor with a psychology degree monitored progress and fidelity, providing mentoring support)	Not reported

Supplementary Table 4: Community Rehabilitation Models in Low-Income Countries (Continued)						
**First author and publication year**	**Name of study model**	**Key components**	**Content of model**	**Setting and country**	**Staffing**	**Duration**
Puspitosari et al. 2019 (37)	Community-based rehabilitation (CBR)	- Comprehensive, multicomponent<br>- Recovery-based<br>- Biopsychosocial approach<br>- Skill-based<br>- Structured psychoeducation<br>- Structured family involvement<br>- Multidisciplinary, task-shared delivery<br>- Culturally adapted (local beliefs, translated content, lay worker delivery)	12 week programme with six psycho-education modules for clients & caregivers (definition, symptoms, management, recovery/relapse, family role, stress management); six social-skills modules for clients (starting a conversation, active listening, making requests, expressing pleasure, expressing displeasure, ending a conversation); interactive methods-discussion, role-play, games, homework-and linkage to ongoing antipsychotic dispensing at the primary-health centre; delivered as weekly group sessions (8–12 participants, 60–90 min)	Community outpatient services, Indonesia	Primary-care physicians, community mental-health nurses, sub-district social-welfare workers, community health workers (*cadres*) (training, ongoing psychiatric supervision)	16 weeks
Chatterjee et al. 2003 (26)	Community-based rehabilitation model (CBR)	- Comprehensive, multicomponent<br>- Recovery-based<br>- Biopsychosocial approach<br>- Skill-based<br>- Structured psychoeducation<br>- Structured family & community participation<br>- Multidisciplinary, task-shared, three-tier delivery Culturally adapted (local beliefs, translated content, lay worker delivery)	Weekly home / village / clinic contacts (60–90 min) by community mental-health workers providing medication-adherence support, psychoeducation, family counselling, vocational & income-generation assistance; fortnightly / monthly village *samiti* meetings to plan rehabilitation & reduce social exclusion; monthly psychiatrist reviews with antipsychotic management (typically risperidone, 2–8 mg)	Community outpatient services, India	Three-tier team: clinic psychiatrist & psychologist; mental health workers with a caseload of 25-30 clients (60-day training) and local village health groups (samitis) involving family members (linked with traditional healers and general practitioners)	12 months

Abbreviations: CBR — Community-Based Rehabilitation; RISE — Rehabilitation Intervention for people with Schizophrenia in Ethiopia

Supplementary Table 5: Illness Management and Recovery Programmes						
**First author and publication year**	**Name of study model**	**Key components**	**Content of model**	**Setting and country**	**Staffing**	**Duration**
Dalum et al. 2018 (28)	Illness Management and Recovery (IMR)	- Integrated (IMR + treatment as usual TAU)<br>- Structured, multicomponent<br>- Recovery-based<br>- Manualised<br>- Goal setting<br>- Structured psychoeducation<br>- Skill-based<br>- Group-based<br>- Family involvement optional	11 modules: recovery strategies, illness education, stress-vulnerability, social support, medication, substance use, relapse prevention, healthy living, stress management, coping, personal goals; weekly 60-min closed-group sessions (max 10); uses CBT and motivational interviewing; personal recovery goals broken into steps; optional family involvement; includes motivational phone calls and in-hospital sessions<br><br>+ TAU by a case manager individually, with weekly meetings at CMHC, home, or other centre activities.	Community outpatient services, Denmark	Two or three mental health professionals per group (minimum 3-day IMR curriculum training, ongoing monthly supervision)	9 months
Roosenschoon et al. 2021 (38)	Illness Management and Recovery (IMR)	- Integrated (IMR + case as usual CAU)<br>- Structured, multicomponent<br>- Recovery-based<br>- Manualised<br>- Goal setting<br>- Structured psychoeducation<br>- Skill-based<br>- Group-based<br>- Structured family and peer involvement	11 modules: recovery strategies, illness education, stress-vulnerability, social support, medication, substance use, relapse prevention, healthy living, stress management, coping, and personal goals; weekly 90-min group sessions (max 8); uses CBT, motivational interviewing, peer support; includes goal setting; homework and workbook assignments provided digitally; family and peer involvement encouraged throughout<br><br>+ CAU: outpatient psychiatric care including biweekly case management, multidisciplinary treatment, and rehabilitation services.	Supported accommodation, Netherlands	Two case managers (mental health nurses or social workers) per group; trained with a minimum 2-day IMR curriculum, plus biannual 4-hour booster sessions and biweekly 2-hour supervision by a senior counsellor.	18 months

Supplementary Table 5: Illness Management and Recovery Programmes (Continued)						
**First author and publication year**	**Name of study model**	**Key components**	**Content of model**	**Setting and country**	**Staffing**	**Duration**
Salyers et al. 2014 (39)	Illness Management and Recovery (IMR)	- Structured, multicomponent<br>- Recovery-based<br>- Manualised<br>- Goal setting<br>- Structured psychoeducation<br>- Skill-based<br>- Group-based<br>- Structured family and peer involvement	10 modules: recovery strategies, illness education, stress-vulnerability, social support, medication, substance use, relapse prevention, healthy living, stress management, coping, and personal goals; weekly group sessions; uses CBT, motivational interviewing; includes goal setting; home assignments; family and peer involvement	Community outpatient, United States	Master's- or doctoral-level clinician + clinical psychology doctoral student; IMR-trained; biweekly 2 hours phone supervision	18 months
Färdig et al. 2011 (30)	Illness Management and Recovery (IMR)	- Structured, multicomponent<br>- Recovery-based<br>- Manualised<br>- Goal setting<br>- Structured psychoeducation<br>- Skill-based<br>- Group-based<br>- Structured family and peer involvement	11 modules: recovery strategies, illness education, stress-vulnerability, social support, medication, substance use, relapse prevention, healthy living, stress management, coping, personal goals; weekly 60-min group sessions (max three or four clients); uses CBT and motivational interviewing; includes goal setting; homework and workbook assignments provided digitally; family and peer involvement	Community outpatient services, Sweden	Two clinicians per group (minimum 5-days MR curriculum training, weekly supervision)	21 months

Supplementary Table 5: Illness Management and Recovery Programmes (Continued)						
**First author and publication year**	**Name of study model**	**Key components**	**Content of model**	**Setting and country**	**Staffing**	**Duration**
Hasson-Ohayon et al. 2007 (33)	Illness Management and Recovery (IMR)	- Structured, multicomponent<br>- Recovery-based<br>- Manualised<br>- Goal setting<br>- Structured psychoeducation<br>- Skill-based<br>- Group-based<br>- Structured family and peer involvement	IMR modules: illness education, goal setting, relapse prevention, coping strategies, and medication adherence; includes goal setting; homework assignments; educational handouts translated into Hebrew and adapted for use in Israel; weekly structured group sessions (60 min each)	Community outpatient services, Israel	Two clinicians per group (psychologists, social workers, occupational therapists); initial structured training (12 sessions × 4 hours each) and ongoing monthly supervision	8 months

Abbreviations: IMR — Illness Management and Recovery; TAU — Treatment as Usual; CAU — Care as Usual; CBT — Cognitive Behavioural Therapy; CMHC — Community Mental Health Centre.

**Supplementary Table 6: Intensive Case Management**						
**First author and publication year**	**Name of study model**	**Key components**	**Content of model**	**Setting and country**	**Staffing**	**Duration**
Sohn et al. 2023 (40)	Seoul-Intensive Case Management (S-ICM)	- Comprehensive, multicomponent<br>- ACT-based approach<br>- Personalised, intensive case management (≤ 5 clients per nurse)<br>- Biopsychosocial approach<br>- Outreach<br>- Structured family involvement<br>- Multidisciplinary delivery	At least weekly home / community contacts providing medication & symptom monitoring; individual counselling; training in daily-living, financial and health-care skills; brokering of psychosocial rehabilitation and social-welfare benefits; work with families on mental-health education; advocacy for community integration. Service plans revised every 3 months; clients step down to standard case-management once goals are met	Community outpatient services, Korea	One dedicated case manager per ≤ 5 clients; caseloads not shared. Each centre's multidisciplinary ICM-committee (psychiatrist, nurses, social workers, psychologists) provides regular supervision and case reviews.	9 months
Fernandez-Miranda et al. 2022 (31)	Case-Managed Programme (CMP)	- Comprehensive, multicomponent<br>- Personalised, intensive case management (≤ 20 clients per nurse)<br>- Integrated pharmacological + psychosocial treatment<br>- Biopsychosocial approach<br>- Outreach<br>- Multidisciplinary delivery	Community-based continuum of care 24-h service offering day hospital, ambulatory and home care; interventions include antipsychotic management (emphasis on LAIs), cognitive remediation, social-skills, self-care training, psychoeducation, vocational support, home assistance; contact tailored to need; at least one clinician visit every 15 days, weekly nurse/case-manager outreach, fortnightly group sessions (50 min) for psychoeducation, cognitive remediation and social skills	Community outpatient services, Spain	Multidisciplinary team: psychiatrists, clinical psychologists, specialist mental-health nurses (case-managers), social workers and occupational therapists; team meets thrice weekly and provides 24-h cover; caseload ≤ 20 per nurse.	10 years

Abbreviations: S-ICM — Seoul-Intensive Case Management; ACT — Assertive Community Treatment; CMP — Case-Managed Programme; LAIs — Long-Acting Injectables; ICM — Intensive Case Management

Supplementary Table 7: Psychosocial Rehabilitation Models						
**First author and publication year**	**Name of study model**	**Key components**	**Content of model**	**Setting and country**	**Staffing**	**Duration**
Ahmed et al. 2021 (22)	Social and Cognitive Rehabilitation Programme	- Structured, multicomponent psychosocial programme<br>- Recovery-based<br>- Skill-based<br>- Intensive, high-frequency schedule (≈ 9 sessions / week)<br>- Structured psychoeducation	Phase 1 (6 sessions on illness insight and engagement); phase 2 (120 sessions consisting of psychoeducation (2–3 h per day, flexible) + cognitive and social-skills 2×/week (60–90 min) + physical exercise 1×/week (45–60 min) + recreation therapy 1–2×/week (≈ 90 min)); interactive group & one-to-one activities aim to build conversation skills, cognitive domains and motivation; ongoing bedside/home-ward practice encouraged	Inpatient setting, Sudan	Nurses and two psychologists	9 months
Arslan et al. 2014 (23)	Psychosocial Rehabilitation (PR)	- Structured, multicomponent psychosocial programme<br>- Recovery-based<br>- Skill-based<br>- Intensive, high-frequency schedule<br>- Structured psychoeducation (clients &family)<br>- Structured family involvement<br>- Multidisciplinary delivery	Focus on therapeutic relationships, stigma reduction, and goal setting; personalised plans developed using comprehensive patient–family rehabilitation form; components- weekly individual consultations (CBT, psychodrama), 14-week psychoeducation (clients & relatives), intensive social skills groups, ongoing interaction groups, optional art/rhythm therapy, and mandatory 3-month workshop participation; progress is regularly reviewed in team meetings; approximately 10.5 hours per week	Community outpatient services, Turkey	Team consultants (psychiatrists & psychologists) (ongoing training, supervision meetings), nurses (some as therapists/assistant therapists), workshop instructors	6 months

Supplementary Table 7: Psychosocial Rehabilitation Models (Continued)						
**First author and publication year**	**Name of study model**	**Key components**	**Content of model**	**Setting and country**	**Staffing**	**Duration**
Wang et al. 2013 (45)	Psychosocial Rehabilitation Training (PsRT)	- Structured, multicomponent psychosocial programme<br>- Skill-based<br>- Structured psychoeducation (clients &family)<br>- Structured family involvement	Four components: psychoeducation, independent living skills, social and vocational skills, cognitive intervention (individual + group, 8–10 participants); family involved in all modules; delivered monthly (2 hrs) for 18 sessions; integrated with routine antipsychotic medication	Community outpatient services, China	Psychiatrists + families act as co-facilitators for homework and monitoring	18 months

Abbreviations: PR — Psychosocial Rehabilitation; PsRT — Psychosocial Rehabilitation Training; CBT — Cognitive Behavioural Therapy
